# BSc-DARTS: Neural Architecture Search for Automatic Diagnosis of Bone Metastases in Bone Scintigrams

**DOI:** 10.2174/0115734056412398251201105549

**Published:** 2026-02-02

**Authors:** Yongchun Cao, Lisen Peng, Qiang Lin, Xianwu Zeng, ZhengXing Man, Caihong Liu, Zhengqi Cai, Xiaodi Huang

**Affiliations:** 1 School of Mathematics and Computer Science, Northwest Minzu University, Lanzhou, China; 2 Gansu Provincial Engineering Research Center of Multimodal Artificial Intelligence, Northwest Minzu University, Lanzhou, China; 3 Key Laboratory of China’s Ethnic Languages and Information Technology of Ministry of Education, Northwest Minzu University, Lanzhou, China; 4 Department of Nuclear Medicine, Gansu Provincial Cancer Hospital, Gansu Provincial Academic Instiute for Medical Research, Lanzhou, China; 5 School of Computing, Mathematics and Engineering, Charles Sturt University, Albury, New South Wales, Australia

**Keywords:** Neural architecture search, DARTS, Bone scintigrams, Image classification, Metastasis detection, Bone metastases

## Abstract

**Introduction:**

Bone scintigram is a highly effective medical imaging technique widely used for the rapid screening of bone metastases, contributing significantly to early disease detection and prognosis assessment. Neural architecture search enables automated design and optimization of network structures by leveraging data characteristics and task-specific requirements.

**Objective:**

For the automated and accurate diagnosis of bone metastasis in bone scintigrams, this study proposes an improved differentiable neural architecture search framework to address two key limitations of the original DARTS method: (1) the insufficient representation capability of standard candidate operations for characterizing bone metastasis lesions, and (2) architecture degeneration.

**Methods:**

Based on the DARTS framework, two novel candidate operations were developed, and the training supernet architecture was optimized. (1) The channel-attention-integrated residual operation incorporates synergistic channel-wise intelligent weighting and gradient stabilization mechanisms, providing an efficient yet highly discriminative feature transformation module for architecture search. (2) The spatial-attention-enhanced multi-branch operation combines multi-scale feature fusion with spatially adaptive focusing, significantly improving the model’s ability to localize critical regions and detect lesions of varying sizes. (3) A dual-path convolutional structure is introduced into the training supernet to dynamically optimize both channel-wise and spatial dimensions of shallow features, thereby generating highly discriminative representations for subsequent cell architecture search.

**Results:**

Comprehensive evaluations on clinical datasets demonstrate the effectiveness of the proposed method, achieving an accuracy of 0.8451, precision of 0.8700, recall of 0.8447, F1-score of 0.8423, and an AUC of 0.92.

**Discussion:**

The results validate that incorporating manual network design experience into the architecture search process can significantly improve model performance. Systematically leveraging anterior-posterior views of the same case can improve lesion detection in complex anatomical regions. Future work will focus on developing a dual-view architecture search framework based on complementary feature fusion.

**Conclusion:**

The proposed neural architecture search method effectively detects bone metastasis in bone scintigrams and outperforms existing state-of-the-art approaches. The newly developed candidate operations and supernet optimizations successfully address the limited representational capacity of standard convolutions in the original DARTS operations, leading to substantial improvements in classification performance.

## 1 INTRODUCTION

Bone metastasis is a frequent complication of solid tumors, including breast, prostate, and lung cancers [1]. Associated symptoms such as bone pain, fractures, hypercalcemia, and spinal cord compression severely impair patients’ survival and quality of life [2]. Therefore, early detection of bone metastases is critical for disease staging, prognosis assessment, and treatment planning. Compared with Positron Emission Tomography (PET), Single Photon Emission Computed Tomography (SPECT) is widely adopted in clinical practice due to its cost-effectiveness and is commonly used as a primary tool for the rapid screening of bone metastases. However, SPECT is characterized by high sensitivity but low specificity [3], and its relatively low spatial resolution enables early lesion detection but poses challenges for precise diagnosis.

Some early studies employed traditional machine learning methods to automatically detect bone metastases by classifying bone scintigrams [4, 5]. However, these methods rely heavily on handcrafted features and lack the ability to automatically learn representations from data, resulting in limited generalization capability.

As a core technology of deep learning (DL), the convolutional neural network (CNN) has achieved great success in computer vision due to its powerful automatic feature extraction capabilities and flexible architecture. CNNs have been widely applied to medical imaging tasks such as classification [6-8], segmentation [9-11], and object detection [12-14]. They have also shown notable advances in detecting metastases of various tumors in nuclear medical images, including prostate cancer [15, 16], breast cancer [17], and lung cancer [18, 19].

Designing deep neural network models for specific tasks often requires the manual construction of core components and architectures, which is time-consuming and dependent on expert knowledge. With increased computing power and growing data complexity, modern network designs exhibit diverse connection patterns and increasingly sophisticated components, further escalating the trial-and-error cost of network development. Neural architecture search (NAS) offers an alternative by automatically constructing optimal network models tailored to specific data and tasks through adaptive adjustment of architectural components.

NAS formulates the exploration of neural architectures as an optimization problem and employs optimization algorithms to solve it [20]. Early NAS approaches primarily used reinforcement learning (RL) or evolutionary algorithms (EAs) as search strategies to identify optimal network structures. The methods in [21, 22] utilize RL, where an agent interacts with the environment and receives rewards that guide subsequent state selections, ultimately converging on the architecture with the highest cumulative reward (*i.e*., the best-performing structure for a given task). Studies in [23, 24] employ EAs, iteratively refining architectures through population-based mutation and recombination to derive optimal solutions. Although these methods can yield high-performance architectures, they often suffer from prohibitively high computational costs.

Architecture search methods leveraging RL or EAs treat neural architecture search as a black-box optimization problem using a discrete search strategy [25]. This approach, which operates over a fixed set of candidate operations, is computationally inefficient. To address this issue, Liu *et al*. [26] proposed Differentiable Architecture Search (DARTS), which relaxes the discrete search space into a continuous domain through a differentiable search strategy. DARTS simultaneously optimizes architectural parameters and network weights using gradient descent, enabling efficient identification of substructures without exhaustive evaluation during the search process.

Based on DARTS, this study proposes a neural architecture search method for automatically detecting bone metastases in bone scintigrams. By fully integrating validated design heuristics from expert-crafted networks, we construct two expressive candidate operations that combine attention mechanisms with residual and multi-branch modules, respectively. This design not only extracts richer image features but also mitigates network collapse issues during architecture search. Additionally, we introduce a dual-path, multi-scale feature extraction module within the supernet training framework to comprehensively learn features at different spatial resolutions, thereby enhancing classification performance.

The main contributions of this work are as follows:

- We introduce a channel attention mechanism into residual modules and integrate spatial attention with multi-branch structures to construct two more competitive candidate operations, effectively expanding the search space.
- We incorporate multi-branch structures and attention mechanisms into the training supernet to facilitate comprehensive extraction of multi-scale features from input images, enhancing channel representation and spatial focus, which improves the final model performance.
- We conduct extensive evaluations on a clinically acquired bone scintigram dataset, demonstrating that our proposed method achieves superior performance compared to existing state-of-the-art approaches.

The rest of this paper is organized as follows: Section 2 reviews related work. Section 3 introduces the experimental dataset used in this study and provides a detailed description of the proposed method. Section 4 presents the experimental results on the bone scintigram dataset. Section 5 discusses the searched cell structures and factors contributing to misclassification. Section 6 outlines limitations and future research directions.

## 2 RELATED WORK

### 2.1 Bone Scintigraphy Classification

In recent years, deep learning methods have emerged in the diagnosis of metastatic lesions in bone scintigraphy. Dang pioneered the application of CNNs for automatic diagnosis of prostate cancer bone metastasis, achieving 89% accuracy and establishing a deep learning-based diagnostic framework [27]. Papandrianos *et al*. [28] enhanced pathological specificity by refining CNNs to effectively differentiate metastatic from degenerative changes. Zhao *et al*. [18] designed a dual-branch ResNet that fuses features from anterior and posterior views, significantly improving multi-view information utilization. Mu *et al*. [29] developed a whole-body scanning diagnostic model for detecting skull base invasion in nasopharyngeal carcinoma, with its generalizability validated through multicenter studies. Furthermore, Morales *et al*. [30] introduced MobileLookNet, a lightweight model using depthwise separable convolutions to optimize computational efficiency, laying the foundation for mobile deployment.

To enhance lesion perception capabilities, attention mechanisms have been widely adopted. Li *et al*. [31] embedded spatial attention modules into a tri-view CNN for lung cancer subtype classification; Lin *et al*. [32] fused channel and spatial attention to amplify responses in lesion regions; Wang *et al*. [33] integrated dual attention with visual analytics to improve decision interpretability; Wei *et al*. [34] proposed an anatomically-aware Transformer that captures long-range dependencies across anatomical regions. Collectively, these advancements leverage multi-view fusion, feature refinement, and adaptive focusing mechanisms, facilitating the transition of bone scan diagnostics toward a high-precision, high-efficiency, and intelligent paradigm.

### 2.2 Neural Architecture Search and Medical Image Classification

Despite reducing search time, DARTS still requires substantial computational resources, especially on large-scale datasets. Moreover, as search iterations increase, the skip-connect operation tends to dominate the final architecture, degrading network performance (*i.e*., collapse) [35]. To address these issues, several studies have proposed diverse improvements. Liang *et al*. [36] introduced an early stopping mechanism to mitigate collapse while enhancing search efficiency. Xu *et al*. [37] proposed partial channel connections to reduce memory and computational costs, alongside Edge Normalization to mitigate selection instability. Chu *et al*. [38] replaced Softmax with Sigmoid for architectural weight processing, enabling multiple operations to coexist at each node to ensure equal opportunities for all operations, thereby reducing the dominance of skip-connects, and introduced an auxiliary loss to counteract performance loss from discretization bias. However, these improvement measures primarily focus on local algorithmic optimizations, addressing the dominance of skip connection dominance through passive suppression rather than through proactive structural redesign (restructuring), which ultimately fails to achieve a fundamental balance between architectural fairness and model performance. Xue *et al*. [39] proposed a gradient-guided evolutionary neural architecture search method that integrates evolutionary algorithms with differentiable search. This hybrid approach achieved low error rates on CIFAR and ImageNet at ultra-low computational cost. Xue *et al*. [40] developed the SWD-NAS algorithm, which employs dual attention mechanisms and architecture weight normalization to effectively resolve performance collapse in differentiable search, significantly outperforming existing gradient-based methods.

NAS has also demonstrated significant efficacy in medical image classification. Wang *et al*. [41] proposed a multi-scale training-free NAS method that outperforms both manually designed networks and traditional NAS approaches on large-scale medical imaging benchmarks and breast cancer datasets. Zechen *et al*. [42] developed PSNAS-Net, which enables an automated search for lightweight yet high-performance models *via* a rule-constrained two-stage search at minimal computational cost. Khan *et al*. [43] integrated Ant Colony Optimization with NAS within a decentralized federated learning framework, attaining 89.7% accuracy in mammography classification.

Despite these advances, the applications of NAS to bone metastasis diagnosis remain limited. Leveraging architecture search to automate network design-capable of adapting to multi-scale characteristics of bone metastatic lesions while optimizing pathological specificity recognition-holds promise for significantly accelerating precision diagnostics and treatment in nuclear medicine imaging.

## 3 MATERIALS AND METHODS

### 3.1 Experimental Dataset

The whole-body bone scintigrams used in this retrospective study were collected from the Department of Nuclear Medicine at Gansu Provincial Cancer Hospital between January 2018 and December 2022. Imaging was performed using a single-head imaging device (GE SPECT Millennium MPR) between 2 and 5 hours after intravenous injection of ^99m^Tc-MDP (20~25 mCi). For each patient, anterior and posterior views were acquired and stored as DICOM format files. Parsing the DICOM files yields bone scintigrams with a resolution of 256 × 1024.

Due to individual differences in the absorption capacity of radioactive tracers, substantial variability exists across patient imaging data. Additionally, bone imaging is susceptible to noise interference from external environmental factors and equipment during acquisition. To enhance the performance of deep classification models, this study employed Z-score normalization to reduce inter-patient variability. Furthermore, an adaptive threshold denoising method was applied to reduce noise in the bone scintigrams.

Since bone metastases occur predominantly in thoracic regions such as the ribs and spine, and considering the training efficiency of the deep model, we extracted a 256 × 256 thoracic region from each whole-body bone scintigram to construct the dataset. The dataset included 504 patients with No Bone Metastasis (NBMet) and 501 patients with Bone Metastasis (WBMet), with both anterior and posterior images for each patient. Thus, the dataset contained a total of 2010 thoracic-region bone scintigrams. The statistics of the dataset are summarized in Table **1**.

### 3.2 The Proposed Method

Based on the DARTS framework, we propose a Differentiable Architecture Search Method for Bone Scintigram Classification (BSc-DARTS) to automatically diagnose bone metastases. Leveraging manual network design expertise, we extend the search space by constructing two enhanced feature-representative candidate operations and employ a dual-path multi-scale convolutional structure to strengthen shallow feature extraction during supernet training. This approach concurrently mitigates the architecture collapse phenomenon inherent in DARTS while significantly improving the performance of the ultimately searched networks.

#### 3.2.1 Search Strategy

In this work, we leverage the DARTS-based search strategy to discover optimal cells as fundamental computational units. The resulting cells are systematically composed in a hierarchical topology, thereby forming the overall classification network. Each cell is conceptualized as a directed acyclic graph (DAG), in which each node corresponds to a potential feature representation, and each directed edge is associated with a learnable operation (*e.g*., convolution, pooling) that transforms the representation of the source node into that of the target node.

During architecture search, the discrete search space is continuously relaxed by replacing the categorical selection of a specific operation between two nodes with a softmax-weighted summation over all candidate operations. A bi-level optimization strategy is employed to alternately update the architecture parameters (on the validation set) and the network weights (on the training set). After the search is completed, the optimal subnetwork is obtained by parsing the architecture parameters, typically by retaining operations with the highest architectural weights.

#### 3.2.2 Search Space Enrichment

The candidate operations included in the search space significantly influence the discovery of task-specific optimal architectural parameters. However, within the DARTS frame-work, a “collapse” phenomenon often occurs when training over a large number of epochs. Specifically, due to biased com-petition among candidate operations during the cell search process, skip-connect operations tend to dominate the network structure, ultimately leading to performance degradation. To mitigate this collapse while still leveraging skip-connect effectively and to enhance the performance of the discovered network we propose two novel candidate operations that integrate attention mechanisms with residual modules and multi-branch structures, respectively. These designs expand the search space and provide richer feature representation capabilities.

##### 3.2.2.1 Res_Separ_SE Candidate Operation

Many manual design experiences of deep neural networks have shown that attention mechanisms can effectively improve the accuracy and efficiency of task processing by focusing on critical information while suppressing irrelevant information. In this study, we integrate channel attention mechanisms with residual structures to construct a novel candidate operation, Res_Separ_SE, as illustrated in Fig. (**1**), enabling BSc-DARTS to automatically discover network structures with enhanced feature discriminability.

As shown in Fig. (**1**), the Res_Separ_SE operation comprises a 3×3 convolution layer and a residual block integrated with the Squeeze-and-Excitation (SE) attention mechanism [44]. The 3×3 convolution adaptively performs downsampling based on the stride inherited from the preceding cell, thereby satisfying the distinct processing requirements of normal cells and reduction cells for feature maps of varying spatial resolutions. The residual block employs 1×1 convolution combined with 3×3 depthwise convolution, which significantly reduces model parameters while maintaining low computational complexity. The integration of the SE attention mechanism enables the searched networks to adaptively learn channel-wise importance weights, followed by recalibrating the feature map through these learned weights. This effectively mitigates the limitation of the insufficient representational capacity found in the original DARTS candidate operations, while providing the search algorithm with an efficient yet highly discriminative feature transformation module. As a result, the accuracy and robustness of the automatically searched network are significantly enhanced for the classification task of Bone Scintigrams.

The SE attention mechanism operates in three stages. First, the Squeeze operation compresses the input feature map into a channel-wise feature vector using Global Average Pooling (GAP) across spatial dimensions. Next, the Excitation step employs two fully connected layers to learn weights for each channel. Finally, in the Scale operation, these learned weights are applied to the original feature map, producing a weighted representation that emphasizes informative channels and suppresses less relevant ones. The mathematical formulation of the SE attention mechanism is presented in Eq. (**1**).

**Table:** 

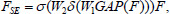	(1)

where *F* denotes the input feature map, *F_SE_* represents the output feature map, *GAP* is Global Average Pooling, *W*_1_ and *W*_2_ correspond to fully connected layers, while *δ* and *σ* represent the ReLU and Sigmoid activation functions, respectively.

##### 3.2.2.2 Asy_Multi_SA Candidate Operation

Multi-branch structure, through heterogeneous branch design, achieves dynamic complementarity between local details and global semantics. Compared with dilated convolution that expands receptive fields *via* sparse sampling, this structure is more effective in capturing subtle metastatic lesions and heterogeneous metabolic regions in bone scintigrams. This study synergistically integrates multi-branch structure with spatial attention (SA) mechanisms to construct a novel candidate operation termed Asy_Multi_SA.

As depicted in Fig. (**2**), the Asy_Multi_SA candidate operation is primarily composed of a 3×3 convolution, a Spatial Attention (SA) module, and a multi-branch structure. The 3×3 convolution adaptively performs downsampling based on the stride inherited from the preceding cell. The integration of the SA module enables the model to adaptively learn positional information of critical regions and perform feature map reweighting based on the learned attention weights. This enables precise localisation of lesion regions within complex backgrounds, effectively addressing the inherent “global average response” bias associated with traditional convolutional operations. Furthermore, the multi-branch structure captures multi-scale features through heterogeneous receptive fields, which strengthens the recognition capability for lesion regions of varying sizes.

The SA module initially performs both average pooling and max pooling operations along the channel dimension of the input feature map. These two pooled feature maps are subsequently concatenated and processed through convolutional layers to progressively fuse and extract spatial information. Finally, a spatial attention weight map is generated *via* the Sigmoid activation function, achieving feature recalibration across distinct spatial locations. The formal mathematical representation of the SA attention mechanism is provided in Eqs (**2**) and (**3**).

**Table:** 

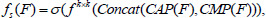	(2)

**Table:** 

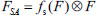	(3)

Where *σ* denotes the Sigmoid activation function. *CAP* and *CMP* represent average pooling and max pooling operations along the channel dimension, respectively. *Concat* indicates feature map concatenation operator, *f ^k^*^×^*^k^* corresponds to a convolution operation with kernel size *k*×*k*, the symbol 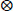 denotes element-wise multiplication, *fs* (*F*) denotes spatial attention weight map, and *F_SA_* represents the output feature map.

In the original DARTS framework, the skip-connect operation performs a simple identity mapping. While this helps alleviate the vanishing gradient problem, it cannot transform features, resulting in insufficient representation of the complex patterns found in bone scintigraphy. In contrast, the residual structures not only preserve identity mapping but also incorporate learnable convolutional layers, thereby enhancing the model’s capacity for feature extraction. Furthermore, it provides a flexible framework for integrating attention mechanisms (*e.g*., SE blocks), which amplify lesion-relevant channel responses. Accordingly, this study retains seven original candidate operations from the DARTS framework while excluding the skip_connect operation. By incorporating two novel self-constructed operations (Res_Separ_SE and Asy_Multi_SA), the search space of BSc-DARTS comprises nine candidate operations: max_pool_3×3, avg_pool_3×3, sep_conv_3×3, sep_conv_5×5, dil_conv_3×3, dil_conv_5×5, Res_Separ_SE, Asy_Multi_SA, and zero.

#### 3.2.5 Architecture Search Training

The training supernet of BSc-DARTS adopts a structure similar to DARTS, where a number of cells are stacked into the overall architecture of the neural network through a derived chain-like structure, and an architectural search is performed within individual cells. The training supernetwork comprises two types of cells: normal cells and reduction cells. All normal cells maintain consistent dimensions between input and output feature maps, whereas reduction cells perform downsampling, halving the spatial dimensions of input feature maps. Each cell has two inputs and one output: the inputs are derived from the outputs of the two preceding layers of cells, and the output is the concatenation of feature maps from all intermediate nodes within the cell. The overall architecture of the BSc-DARTS training supernet is illustrated in Fig. (**3**).

As shown in Fig. (**3**), the training supernet of BSc-DARTS consists of three components: input image feature extraction, cell stacking, and classifier. First, a dual-path convolutional structure is applied to adequately extract features from the input image, generating feature maps. These feature maps are then fed as inputs to the cells, where the optimal substructure of the cells is searched within the search space. Finally, the output feature maps of the cells are integrated through the classifier to produce the final classification results.

The dual-path convolutional structure is designed to adapt to the characteristics of varying sizes and locations of bone metastasis lesions in bone scintigrams. It employs a 3×3 convolutional layer and a multi-branch structure to comprehensively extract multi-scale features from the input image. Additionally, the CBAM [45] attention mechanism is introduced to enhance channel-wise feature representation and reinforce the focus on spatially critical information within feature maps. As a feature refinement module, CBAM is embedded at the terminal position of the dual-path structure. It performs dual-dimensional dynamic optimization along both channel-wise and spatial attributes in shallow features. This dual-dimensional attention mechanism enables the generation of high-quality, lesion-discriminative feature representations, which serve as a more informative input for subsequent cell architecture search. The structure of CBAM is illustrated in Fig. (**4**).

The CBAM attention mechanism integrates channel attention and spatial attention mechanisms, enabling the network to adaptively recalibrate feature map weights, thereby emphasizing critical features and spatial regions of interest. This enhances the feature representation capability of input images. The CBAM comprises two key components: a channel attention module and a spatial attention module, as formalized in Eq. (**4**).

**Table:** 

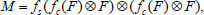	(4)

where *F* denotes the input feature map, 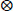 represents element-wise multiplication, *fc*(·) and *fs*(·) correspond to the channel attention and spatial attention functions, respectively, as defined in Eqs. (**2** and **5**).

**Table:** 

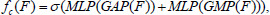	(5)

where *σ* denotes the Sigmoid function, *MLP* refers to the Multilayer Perceptron, *GAP* and *GMP* represent Global Average Pooling and Global Maximum Pooling, respectively.

Given the low-resolution nature of the bone scintigram and the limited dataset size, the backbone network in this study is constructed by stacking 8 normal cells and 2 reduction cells.

## 4 EXPERIMENTS AND RESULTS

In this section, we experimentally evaluate the proposed method using a clinical bone scintigram dataset, as presented in Table **1**.

### 4.1 Experimental Setup

The experimental evaluation metrics used in this work include *Accuracy*, *Precision*, *Recall*, and *F*-1 *score*, as defined in Eqs. (**6**-**9**).

**Table:** 

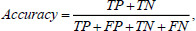	(6)

**Table:** 

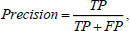	(7)

**Table:** 

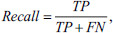	(8)

**Table:** 

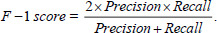	(9)

Where *TP*, *TN*, *FP*, and *FN* denote True Positive, True Negative, False Positive, and False Negative, respectively.

The experimental process employs a randomized approach to partition the dataset. The training set and test set are allocated in a 7:3 ratio, with the anterior and posterior views of the same patient in the same dataset. During the architecture search phase, the training set and validation set are divided into a 5:5 ratio to search for the optimal cell structure. To mitigate the effects of randomness, the experimental results are reported as the average of five independent runs under identical conditions. The parameter settings for the BSc-DARTS method are listed in Table **2**.

The experimental operating environment consists of an AutoDL server equipped with a 15-core Xeon(R) Platinum 8358P processor, 80 GB of RAM, and an A40 GPU, using PyTorch 1.11.0 as the deep learning platform.

### 4.2 Experimental Results

#### 4.2.1 Performance Results

Experiments were conducted on the dataset listed in Table **1** to validate the performance of the proposed architecture search method on bone scintigrams. The experimental results for the four evaluation metrics are presented in Table **3**.

As shown in Table **3**, BSc-DARTS consistently outperforms DARTS across all classification metrics, with significant improvements. Notably, enhancements exceeding 12% are observed in the critical metrics of Recall and F-1 score. The elevated Recall value indicates a substantial reduction in the risk of missed detection for potential lesions in medical imaging, while the remarked improvement in F-1 score demonstrates a better balance between sensitivity and specificity in classification outcomes. By reinforcing representation learning of data characteristics and enhancing the robustness of architecture search, BSc-DARTS exhibits superior clinical utility in bone scintigram classification tasks.

The ROC curves and AUC values corresponding to the optimal network structures searched by DARTS and BSc-DARTS are illustrated in Fig. (**5**).

As shown in Fig. (**5**), the ROC curves illustrate the superior classification performance of BSc-DARTS, which achieves an AUC value of 0.92 compared to 0.87 for DARTS. This significant improvement highlights the enhanced sensitivity and specificity of BSc-DARTS.

**Fig. (6):**
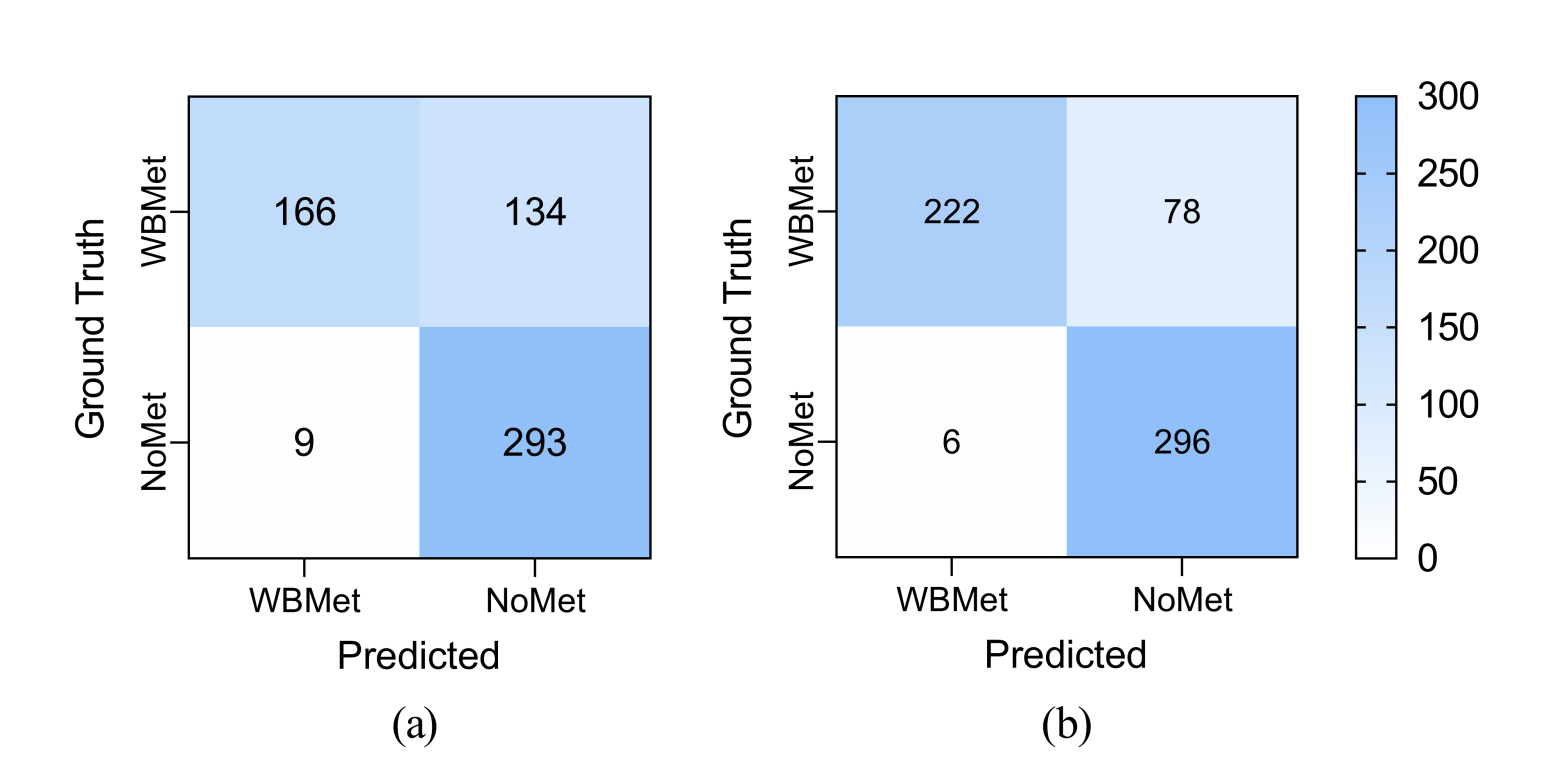
Comparison of confusion matrices for bone scintigrams classification: (**a**) Confusion matrix of DARTS; (**b**) Confusion matrix of BSc-DARTS.

Figure **6** presents the confusion matrices of the DARTS and BSc-DARTS methods on the test set.

As shown in Fig. (**6**), compared to the DARTS, the BSc-DARTS demonstrates significant improvements by reducing both false negatives and false positives on the test samples. These results indicate that BSc-DARTS achieves more accurate and balanced predictions. Moreover, the confusion matrices reveal that the misclassifications primarily arise from false negatives, which will be further analyzed in the discussion section.

#### 4.2.2 Ablation Study

We evaluated the impact of the expanded candidate operation set on classification performance using both the DARTS and BSc-DARTS methods, and the experimental results are presented in Table **4**. In Table **4**, the original candidate operations set of DARTS is labeled as “Original”, while the extended candidate operations set constructed in our BSc-DARTS is labeled as “Extended”.

As shown in Table **4**, the optimal network structures searched with the extended candidate operation set exhibit superior classification performance for both DARTS and BSc-DARTS. These results demonstrate that the introduced Res_Separ_SE and Asy_Multi_SA operations effectively enhance feature modelling capabilities. Furthermore, they indicate that the extended candidate operation set is better suited for bone scintigram classification tasks compared to the original set.

Using the extended candidate operation set, we further investigated the impact of training supernet architecture on classification performance. The corresponding experimental results are summarized in Table **5**.

As shown in Table **5**, incorporating dual-path convolutional operations and the CBAM attention mechanism into the training supernet architecture achieves the best classification performance. This improvement can be attributed to the multi-branch structure of the dual-path convolution, which enables a more comprehensive extraction of multi-scale features from input images. In addition, the CBAM mechanism further enhances feature representation through adaptive channel-spatial attention, thereby significantly enhancing the model's classification performance.

#### 4.2.3 Comparative Analysis

To further evaluate the effectiveness of the proposed method, a comparative analysis was conducted using experimental results from manually designed classic deep models, including GoogLeNet [46], DenseNet121 [47], VGG16 [48], AlexNet [49], and ResNet-18 [50], as illustrated in Fig. (**7**).

As shown in Fig. (**7**), the proposed BSc-DARTS achieves the highest scores across all evaluation metrics, demonstrating that its searched optimal network structure delivers superior classification performance. These results validate the feasibility and effectiveness of the method for bone scintigraphy classification tasks. Further, the experimental results in Fig. (**7**) confirm that architecture search methods can automatically discover optimal structures by exploring search spaces tailored to specific tasks, reducing dependence on designer expertise and trial-and-error costs while outperforming manually designed deep neural networks.

To further validate the performance of the proposed BSc-DARTS in bone scintigram classification tasks, we conducted a comprehensive comparative analysis with state-of-the-art DARTS variants, including PC-DARTS [37], DARTS+ [36], and Fair-DARTS [38]. The experimental results are shown in Fig. (**8**).

As illustrated in Fig. (**8**), all DARTS-based variants exhibit systematic performance improvements over the original DARTS method on the bone scintigram dataset. Notably, the proposed BSc-DARTS achieves statistically significant superiority across all four evaluation metrics, confirming its clinical applicability as a disease-specific neural architecture search framework for nuclear medicine image analysis.

However, as shown in Table **6**, BSc-DARTS exhibits notable disadvantages in terms of parameter efficiency and training time. Due to the integration of manually designed, coarse-grained structural modules as candidate operations, the resulting classification network has a significantly larger parameter size compared to other methods and incurs relatively higher training time costs.

## 5 DISCUSSION

In this section, we concisely discuss three key aspects: (1) the cell structure of the optimal network discovered by BSc-DARTS, (2) a comparative analysis of DARTS-based variants, and (3) the primary causes of misclassification in bone scintigrams.

### 5.1 Analysis of Cell Structures

The cell structures searched by DARTS and BSc-DARTS on the bone scintigram dataset are shown in Figs. (**9** and **10**), respectively.

As shown in Fig. (**10**), in the cells of the optimal network structure searched by BSc-DARTS, the custom-designed candidate operations Res_Separ_SE (The operation indicated by the red arrows) and Asy_Multi_SA (The operation indicated by the Blue arrows) are frequently selected as inter-node operations. Fig. (**10**) shows that these operations account for 3/8 and 2/8 of the inter-node connections in the normal and reduction cells, respectively. Their preferential selection by BSc-DARTS highlights superior effectiveness compared to standard operations (*e.g*., conventional convolutions/pooling) in medical imaging tasks, thereby contributing to superior classification performance of the discovered network structure.

### 5.2 Comparative Analysis of BSc-DARTS and DARTS-based Variants

**Fig. (8):**
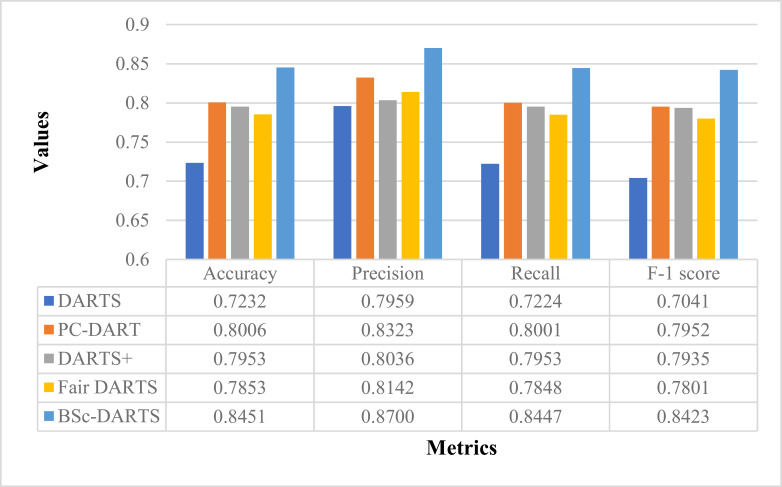
A comparison of classification performance among DARTS-based variants.

Figure **8** presents the classification performance of various DARTS-based variants on the bone scintigram dataset, where the proposed BSc-DARTS method achieves superior performance. We further analyze the underlying mechanisms contributing to this outcome.

The three DARTS-based variants - PC-DARTS, DARTS+, and FairDARTS - primarily focus on optimizing DARTS' search strategy. These improvements aim to enhance search efficiency by reducing memory and computational costs or mitigating performance “collapse”, thereby maintaining or marginally improving model performance. In contrast, BSc-DARTS effectively integrates accumulated expertise from manual network design practices by incorporating residual structures, attention mechanisms, and multi-branch structures -all widely validated in deep learning - into its candidate operations or training supernet. This significantly strengthens the feature extraction capabilities of the searched architectures, enabling BSc-DARTS to surpass other DARTS variants on bone scintigram datasets. These results empirically validate that judicious integration of manual network design experience constitutes an effective approach for enhancing performance in neural architecture search. It is noteworthy that the integration of manually designed coarse-grained modules as candidate operations in BSc-DARTS results in a classification network with a relatively large parameter size.

### 5.3 Analysis of Misclassification Causes

Figure **6** shows that among all 84 misclassified samples, false negatives constituted the vast majority- 92% (78 cases). To further investigate, these 78 false negative samples were further categorized based on lesion site: rib, spine, and multiple lesions. The distribution analysis revealed that false negatives in the rib region accounted for 33 cases (42.3%), the spine region for 29 cases (37.2%), and multiple lesions for 16 cases (20.5%).

**Fig. (11):**
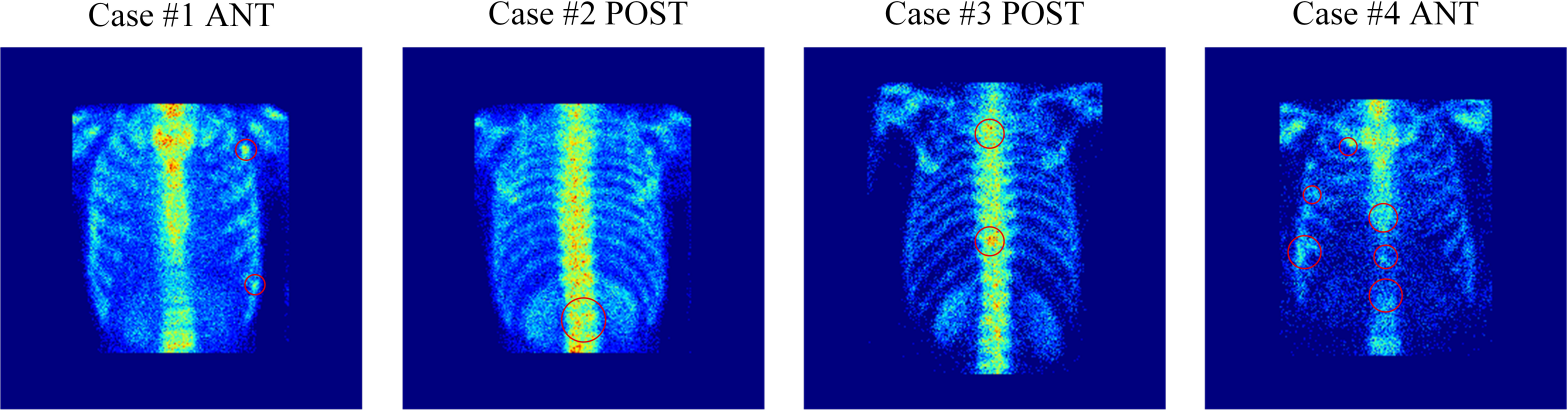
Representative heatmaps of undetected malignant lesions in bone scintigrams predicted by BSc-DARTS.

The main reasons for misclassification varied by anatomical regions:

*Rib region*: Errors were mainly due to the small size of lesions, *e.g*., (Fig. **11**), Case #1. The punctuate or micronodular morphology of these lesions often leads to insufficient local feature extraction.

*Spine region*: Lesions were frequently obscured by normal bone metabolic signals, *e.g*., (Fig. **11**), Cases #2 and #3, making it challenging for the model to distinguish pathological areas.

*Multiple lesions*: False negatives were primarily caused by either small lesion size or metabolic activity levels similar to adjacent tissues, *e.g*., (Fig. **11**), Case #4.

Beyond these inherent limitations of bone scintigrams, the restricted sample size and lack of diversity in the dataset further constrained the model’s generalization, contributing to the observed misclassification.

## 6 LIMITATIONS OF THE STUDY

Although the proposed method demonstrates significant advantages in classification performance, it still faces several limitations and challenges. Firstly, the classification model discovered by BSc-DARTS has an excessively large parameter size, resulting in high resource consumption during practical deployment. Secondly, due to the small-scale dataset and limited sample diversity, the model’s generalization capability remains insufficient, and its ability to distinguish complex lesions requires further improvement. Finally, although fully exploiting data characteristics and incorporating domain knowledge is an effective strategy for enhancing bone metastasis identification, integrating these elements into the NAS search space design remains challenging.

Future work will focus on three main directions:

(1) Developing lightweight operations and optimizing adaptive search strategies to improve BSc-DARTS’s architecture search efficiency and model generalization capability;

(2) Constructing a repository of hard-case lesions to support synthetic data generation for small lesions and overlapping regions, thereby improving the search space’s sensitivity to fine-grained features; and

(3) Systematically utilizing anterior–posterior views of the same case to develop an architecture search framework based on complementary feature fusion of dual-view data, aiming to further enhance lesion detection in complex anatomical regions.

## CONCLUSION

This study proposes a novel neural architecture search framework for bone scintigram classification. By embedding channel attention and spatial attention mechanisms into residual modules and multi-branch structures, respectively, we introduce two enhanced candidate operations with superior representational capacity. This design effectively mitigates the limitations of standard convolutions in the original DARTS-namely, insufficient feature extraction and architectural degeneration induced by skip connections. The dual-path convolutional structure integrated into the training supernet enables dynamic co-optimization of both channel and spatial dimensions in shallow features, producing highly discriminative, lesion-relevant representations for subsequent cell architecture search.

Evaluations on a clinical bone scintigram dataset demonstrate that our method significantly outperforms existing models across key metrics: accuracy (0.8451), precision (0.8700), recall (0.8447), F1-score (0.8423), and AUC (0.92). These results validate its effectiveness and superiority in the automated diagnosis of bone metastases. However, a limitation of the proposed method is its relatively large number of model parameters.

## Figures and Tables

**Fig. (1):**
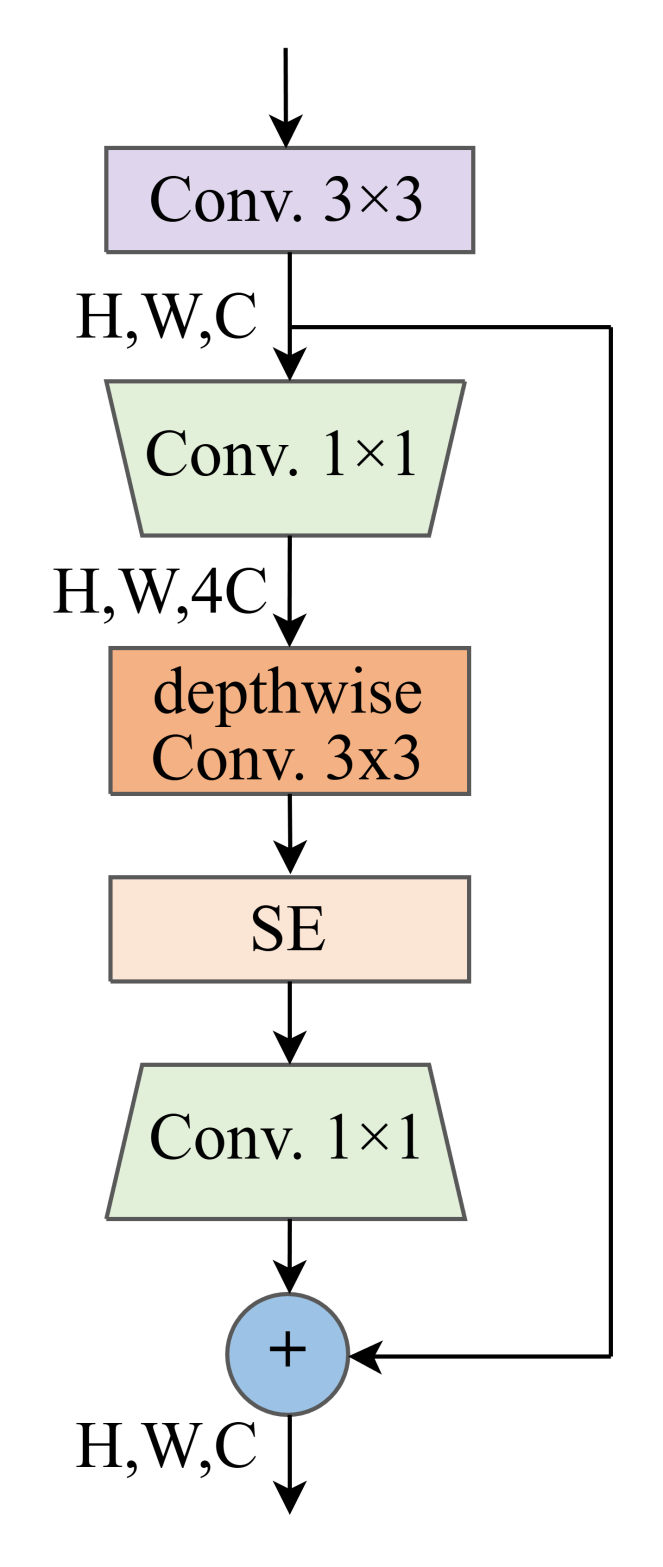
Schematic of the Res_Separ_SE candidate operation integrating channel attention mechanisms with residual depthwise convolution.

**Fig. (2):**
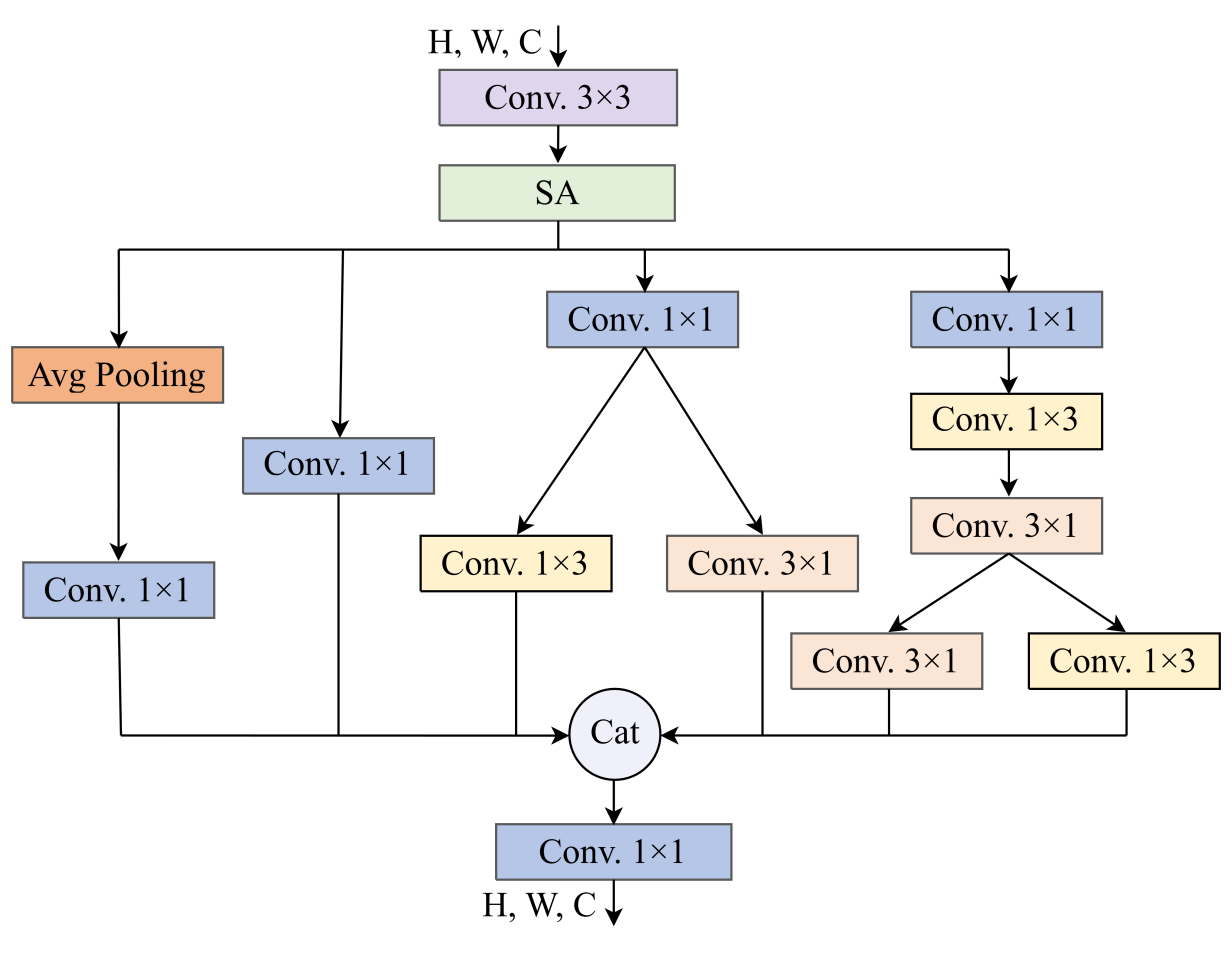
Schematic of Asy_Multi_SA candidate operation integrating spatial attention with a multi-branch structure.

**Fig. (3):**
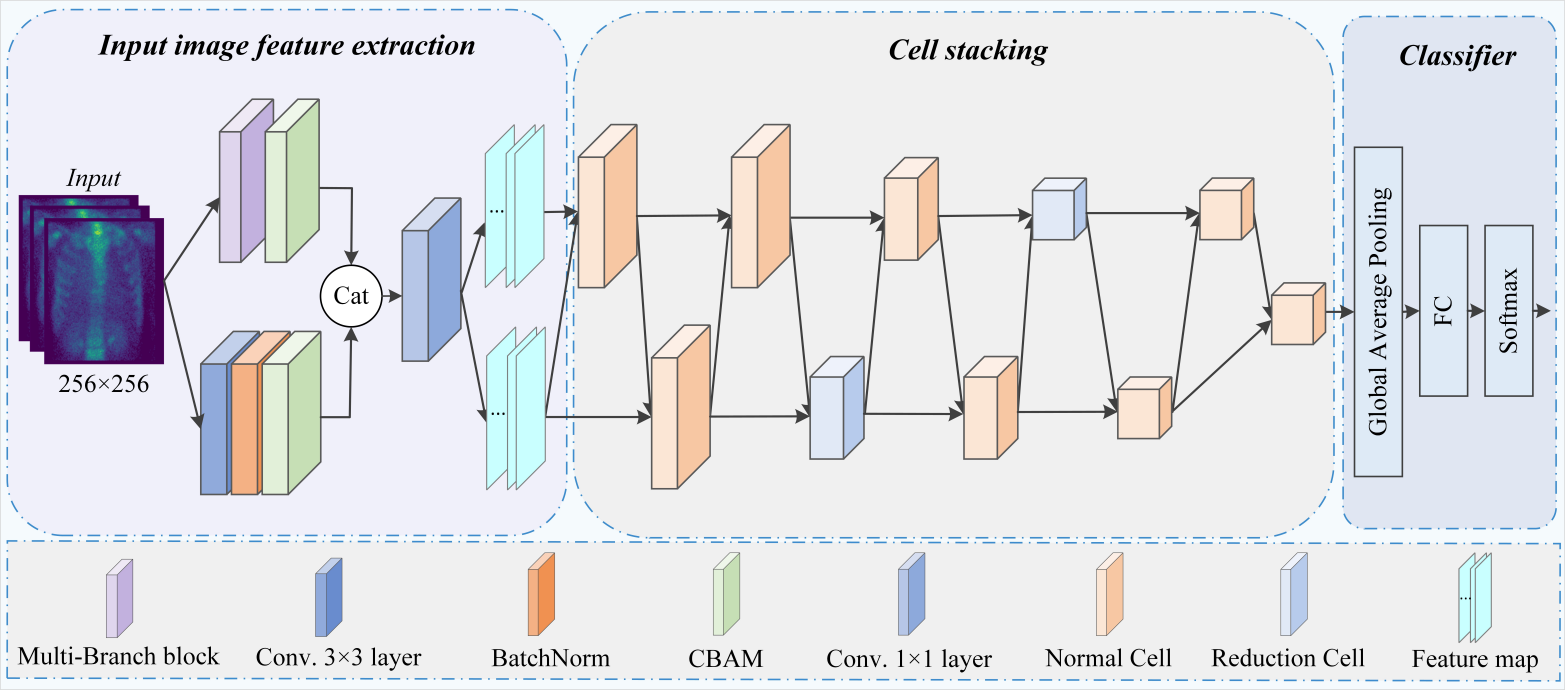
Schematic diagram of the BSc-DARTS supernet training framework for bone scintigrams classification.

**Fig. (4):**
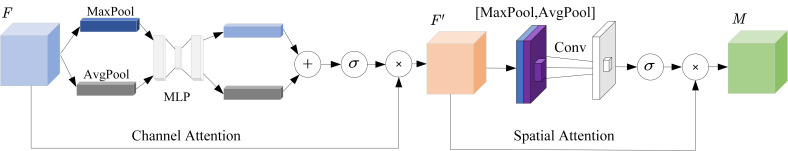
Schematic of the CBAM attention module integrated in BSc-DARTS.

**Fig. (5):**
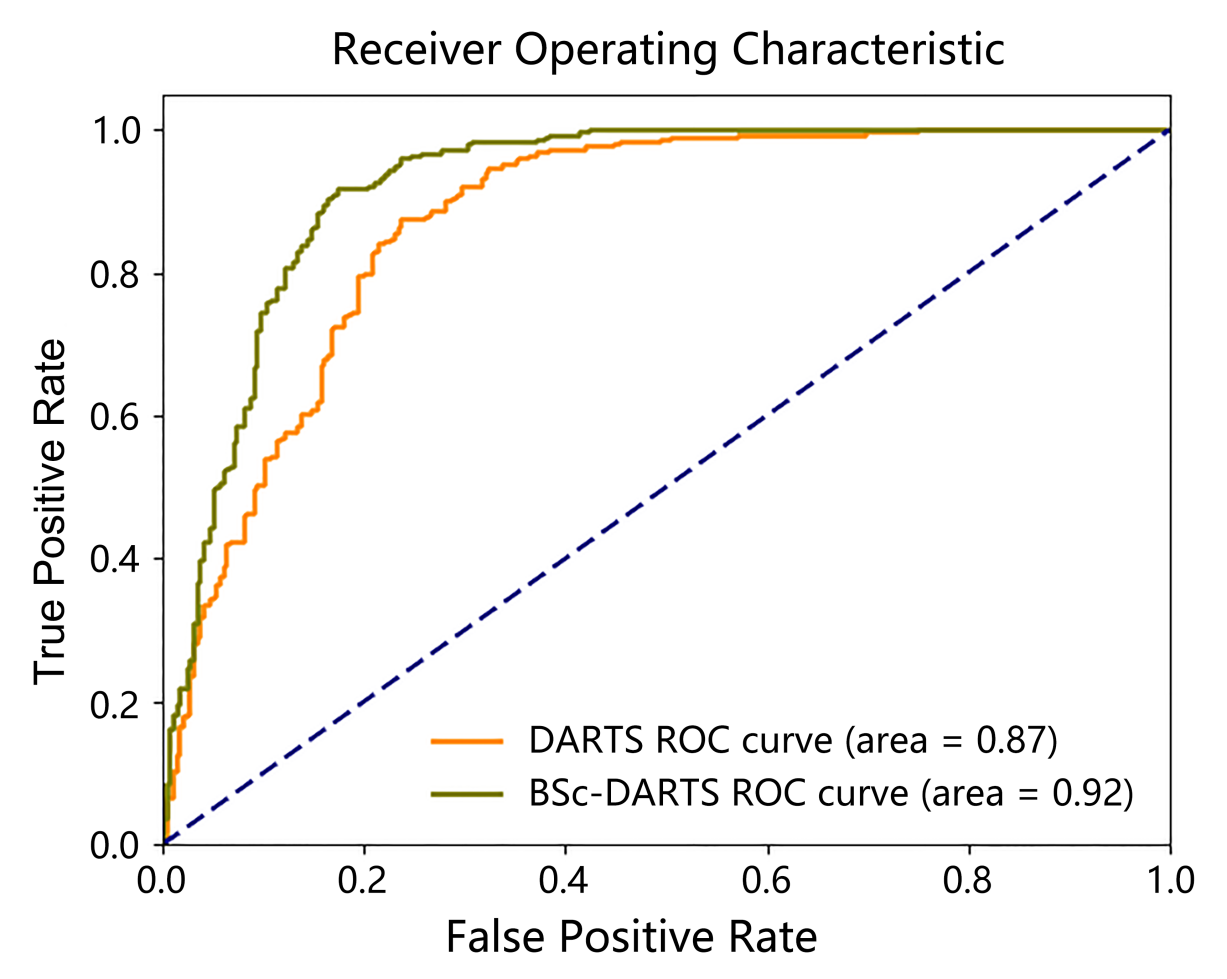
The ROC curves and AUC values correspond to the optimal network structures searched by DARTS and BSc-DARTS.

**Fig. (7):**
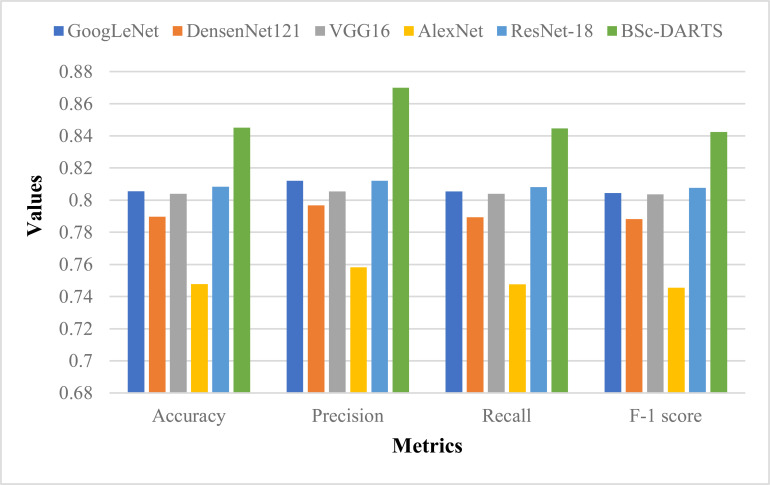
Comparison of BSc-DARTS with existing manually-designed classical classification models.

**Fig. (9):**
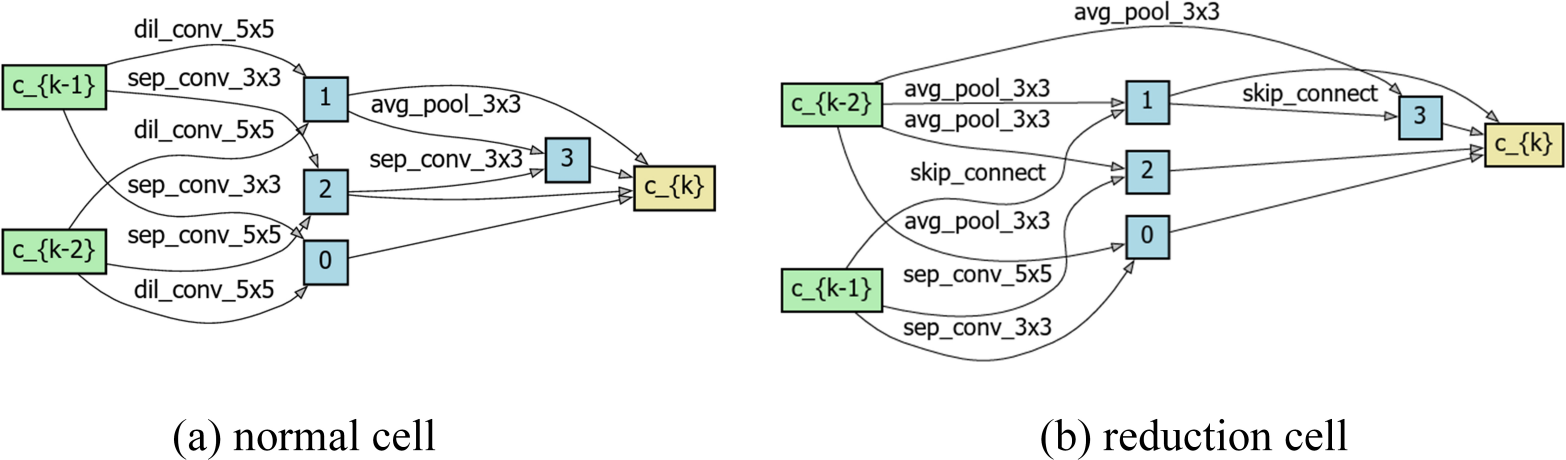
The cell structures searched by DARTS on the bone scintigram dataset.

**Fig. (10a, b):**
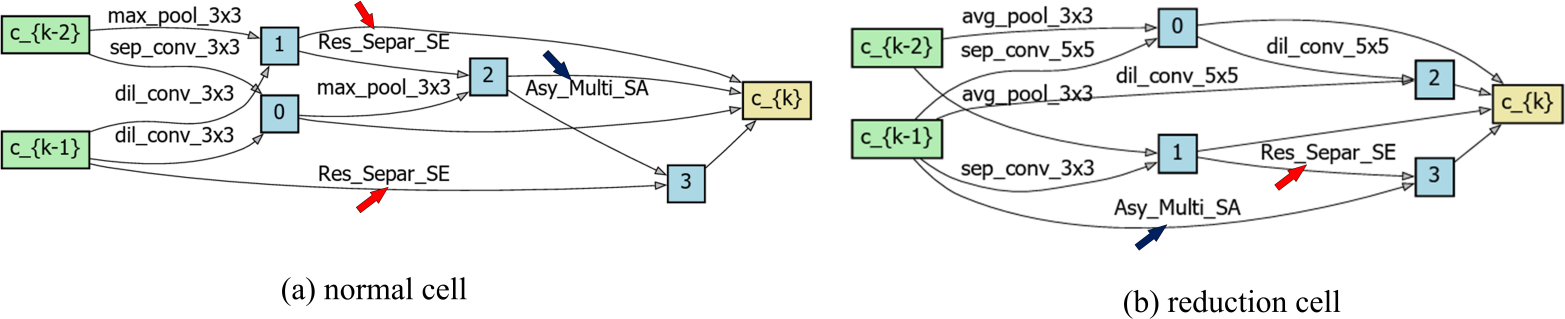
The cell structures searched by BSc-DARTS on the bone scintigram dataset.

**Table 1:** Statistics of the dataset used in this study.

**View**	**NoMet**	**WBMet**	**Total**
Anterior	504	501	1005
Posterior	504	501	1005
Total	1008	1002	2010

**Table 2:** Experimental parameter settings of the BSc-DARTS method.

**Parameters**	**Values**
Learning rate	0.025
Weight decay	0.0003
Batch size	16
Epoch Optimizer Init_channels	200 SGD 8

**Table 3:** The evaluation metric scores of BSc-DARTS and DARTS on the test samples.

**Search Architecture**	**Accuracy**	**Precision**	**Recall**	**F-1 score**
DARTS	0.7232	0.7959	0.7224	0.7041
BSc-DARTS	**0.8451**	**0.8700**	**0.8447**	**0.8423**

**Table 4:** Impact of candidate operation set on classification performance.

**Candidate Operations Set**	**Architecture**	**Accuracy**	**Precision**	**Recall**	**F-1 score**
Original	DARTS	0.7232	0.7959	0.7224	0.7041
Extended	DARTS	0.8345	0.8377	0.8343	0.8341
Original	BSc-DARTS	0.8245	0.8527	0.8241	0.8207
Extended	BSc-DARTS	**0.8451**	**0.8700**	**0.8447**	**0.8423**

**Table 5:** Impact of training supernet architecture on classification performance.

**Dual-path Convolutional**	**CBAM**	**Accuracy**	**Precision**	**Recall**	**F-1 score**
×	×	0.8345	0.8377	0.8343	0.8341
×	√	0.8405	0.8701	0.8400	0.8370
√	×	0.8352	0.8488	0.8350	0.8335
√	√	**0.8451**	**0.8700**	**0.8447**	**0.8423**

**Table 6:** Comparison of parameter sizes and search costs among DARTS-based variants.

**Architecture**	**Params**	**Search Cost (GPU days)**
DARTS	0.064MB	0.179
PC-DARTS	0.011MB	0.233
DARTS+	0.064MB	0.100
Fair-DARTS	0.064MB	0.09
BSc-DARTS	0.568MB	0.195

## Data Availability

The data and supportive information are available within the article.

## LIST OF ABBREVIATIONS

PET: Positron Emission Computed Tomography
SPECT: Single Photon Emission Computed Tomography
DL: Deep learning
CNN: Convolutional neural network
NAS: Neural architecture search
RL: Reinforcement learning
EAs: Evolutionary algorithms
DARTS: Differentiable Architecture Search
GAP: Global Average Pooling

## STANDARDS OF REPORTING

STROBE guidelines were followed.
